# PM2.5-related cell death patterns

**DOI:** 10.7150/ijms.46421

**Published:** 2021-01-01

**Authors:** Yunxia Wang, Yijue Zhong, Jiping Liao, Guangfa Wang

**Affiliations:** 1 Department of Respiratory and Critical Care Medicine, Peking University First Hospital, Beijing, China.; 2 Department of Geriatrics, Jiangsu Provincial Key Laboratory of Geriatrics, the First Affiliated Hospital of Nanjing Medical University, Nanjing, China.

## Abstract

With the increasingly serious problem of environmental pollution, the health problems caused by PM2.5 are gradually coming into our line of sight. Previous researches have indicated that air pollution is nearly related to various diseases, but few studies have focused on the exact function mediated by particulate matter less than 2.5 (PM2.5) in these diseases. PM2.5 is known to induce multiple ways of cell death, including autophagy, necrosis, apoptosis, pyroptosis and ferroptosis. Therefore, it is of much importance to understand the different ways of cell death caused by PM2.5 in the pathogenesis and treatment of PM2.5-related diseases. This present review is an insight of multiple ways of PM2.5‑induced cell death in different diseases.

## Introduction

Previous studies have suggested the conspicuous association between PM2.5 exposure and obviously increased incidence of lots of diseases, such as cardiovascular disease 1, chronic obstructive pulmonary disease 2, Alzheimer's disease 3 and so on. PM2.5 is defined as a harmful type of air fine particulate matter with a diameter of less than 2.5µm 4, characterized by small particle diameter size, large relative surface area and strong toxin absorption ability 5. All these above special characteristics make it easy for PM2.5 to invade into the smallest airways, even the alveolar. PM2.5 particles are mainly composed of solid and liquid, including the main components as black carbon, metal, nitrate, sulfate and polycyclic aromatic hydrocarbons 6. And numerous biological activities have been proved to be challenged by PM2.5, such as cytokine formation and release, coagulation balance, and cardio-pulmonary function 7. And the International Agency for Research on Cancer (IARC) has formally designated outdoor air pollution in general and ambient particulate matter in particular as Class Ⅰ human carcinogens 8. Therefore, it is particularly important to understand the changes of cell fate induced by PM2.5 exposure, so as to find the relevant reversal methods.

Researches have fully indicated that PM2.5 may trigger cell death, including autophagy, necrosis and apoptosis 9-11. Not only that, PM2.5 related pyroptosis and ferroptosis is also been explore in this review.

## PM2.5 and autophagy

Autophagy is known as a physiological subcellular degradation process, including the decomposition of folded proteins, protein complexes and dysfunctional organelles 12. These cytoplasmic component are then sequestered into autophagosomes, as double-membrane vesicles, which are subsequently fused with lysosomes to form autolysosomes and are further degraded into micromolecule by lysosomal hydrolases 13. Autophagy has been regarded as an adaptive response to multiform stimulations, maintaining the dynamic cellular homeostasis, while it was also be activated by several pathogenic and diseases conditions, as well as the execution of a caspase independent cell death 14, 15.

Numerous researches have focused on PM2.5 induced autophagy in different cells or tissues, and multitudinous pathways have been verified to be involved into this autophagy process. PM2.5 can cause autophagy in multiple systems and organs in whole body and participate in the occurrence and development of many diseases, and the respiratory system bears the brunt. Xu et al. revealed that PM2.5 could induce autophagy-mediated cell death through activating NOS2 signaling in human bronchial epithelium cells 16. Qian et al. and Ding et al. found PM2.5 stimulation induced autophagy through ROS pathway in lung cancer cells 17, 18. And Song et al. also showed PM2.5 could induce autophagy via ATR-CHEK1/CHK1 axis, which subsequently activated TP53-dependent autophagy and VEGFA production in BEAS-2B cells 19. Liu et al. indicated that in A549 cells, AMPK was essential for PM2.5-induced autophagy 20. In the reproductive system, Liu et al. showed PM2.5 could induce developmental toxicity through endoplasmic reticulum stress in zebrafish embryos 21. While Wei et al. indicated PM2.5 exposure could damage the integrity of blood-testis barrier through autophagy 22. In the digestive system, inhalation exposure to PM2.5 could induce hepatic cells autophagy in a manner depending on the MyD88-mediated inflammatory pathway in mice 23. Speaking of the circulatory system, PM2.5 induced aortic endothelial cells autophagy by activating the NF-κB pathway in mice 24. In addition to the main system, PM2.5 could also trigger autophagy in human corneal epithelial cell 25.

**Figure 1:**
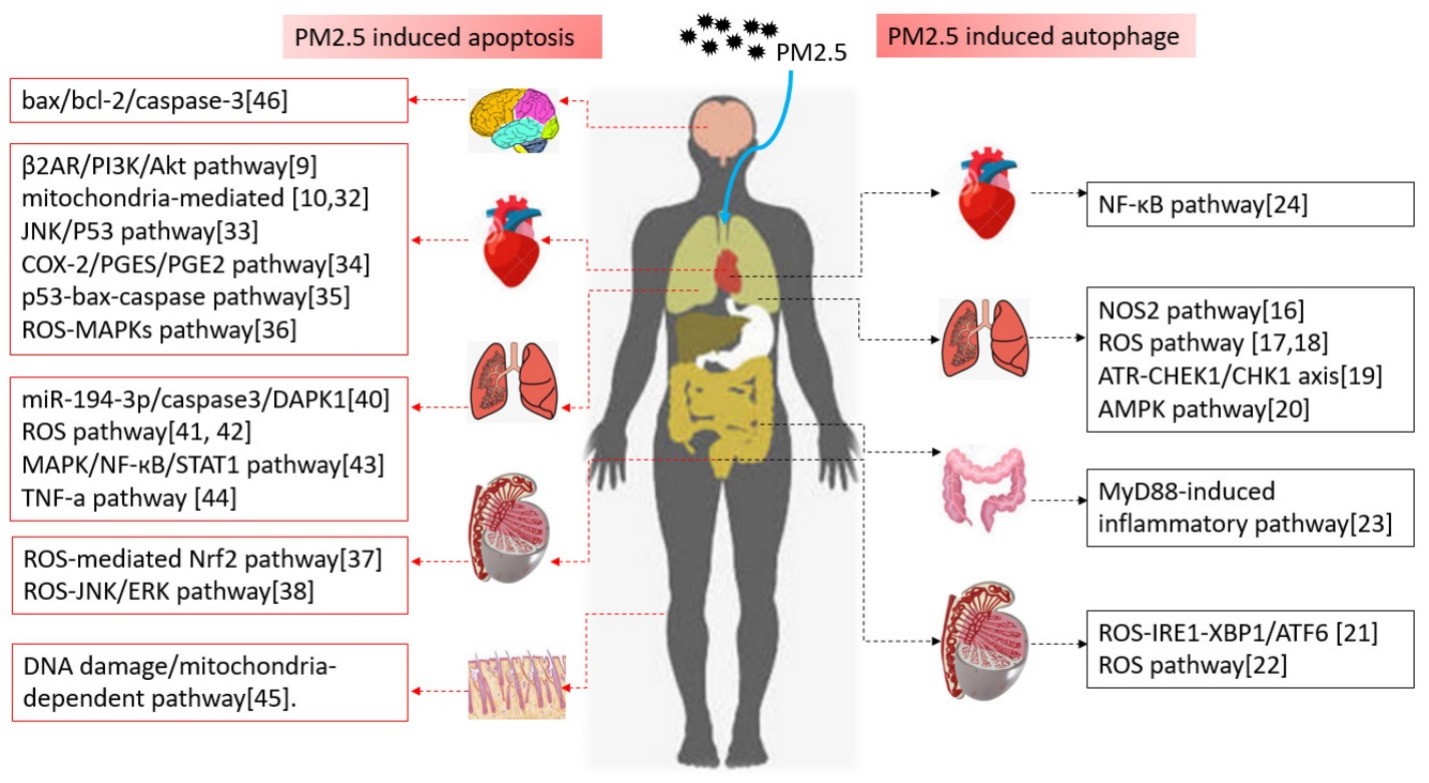
Autophagy, apoptosis and the related signal pathways induced by PM2.5 in different systems

Above all, PM2.5-induced autophagy occurs in all systems, and most of them are involved in oxidative stress and inflammatory signaling pathway. As the above, NOS2 signaling, ROS pathway, AMPK, MyD88-mediated pathways were all involved in PM2.5 induced autophagy (shown in Figure 1).

## PM2.5 and necrosis

Necrotic cells manifest cell swelling and loss of cell membrane integrity, which leads the release of cytoplasmic contents to the surrounding tissue and activation of acute inflammation 26. And necrosis was initially regarded as an accidental form of cell death by acute cellular injury which could induce lethal consequences, but recently, researchers have evidenced that necrosis might be another form of programmed cell death 27. Long term chronic PM2.5 pollutants exposure of endothelial cells from the airways and alveoli could induce various cytotoxic effects, such as oxidative damage and even cell death.

Necrotic cells and neutrophils with co-exudation release lysosomal enzymes. It can promote the further occurrence of necrosis and local parenchyma cell dissolution, so necrosis often involves multiple cells at the same time, and causes tissue inflammation, eventually leading to more serious consequences. PM2.5 first enters the respiratory system and cause related necrosis, so we are more concerned about respiratory damage. Peng et al. stimulated human lung epithelial cells to high doses of PM2.5 (about 400 μg/mL) for about 24h to revealed that PM2.5 could induce cell necrosis in human respiratory system 11. And the PM2.5 induce necrosis in above research was mediated by autophagy. Cell necrosis is caused by oxidative stress exceeding the antioxidant defense capacity of cells. Extremely high dose of PM2.5 may induce oxidative damage to organelles and biological molecules in the meantime, then over exceeded the capacity of cells' autophagy to enter the area of necrosis. In addition to this study, Humar et al. conducted a research to confirm that during an intermediate phase of chronic exposure, PM2.5 triggered the fate changing of cells to maintain energy homeostasis by AMPK pathway, eventually, intracellular PM2.5 accumulation promoted lysosome instability and cell death through lysosomal hydrolases and p38 MAPK. And TEM images revealed the finally status of PM2.5 stimulated BEAS-2B cells was necrosis 28.

Because the first deposit of PM2.5 is the respiratory system, while PM2.5 could not achieve instantaneous high dose accumulation in other systems, the studies focus on cell necrosis caused by PM2.5 mainly in the respiratory system. However, the effect of high concentration of PM2.5 induced necrosis in other systems still needs exploration and attention.

## PM2.5 and apoptosis

Apoptosis plays an important role in animal development and function establishment, such as nonfunctional cells (nonfunctional nerve cells, and activated lymphocytes) elimination and mammary glands involution 29. Apoptosis involves two mechanisms: killing cells mediated by caspase3 or 7; and recruiting macrophages for cell engulfment 30, 31.

Apoptosis induced by PM2.5 involved in various system of the body, as described below. In the circulatory system, Sun et al. found that PM2.5 exposure resulted in cardiomyocytes apoptosis and cardiac dysfunction through the hypermethylation of myocardial ADRB2 and activation of β2AR/PI3K/Akt pathway 9. And they also demonstrated that mitochondria-mediated apoptosis pathway played a critical role in PM-induced myocardial cytotoxicity in AC16 contributing to cardiac dysfunction 32. As is also in myocardial cells, they found that PM2.5 aggravated mitochondrial damage, lipid accumulation and apoptosis in macrophage foam cells to induce atherosclerotic plaque progression 10. Apart from Prof. Sun's researches, Xu et al. found that PM2.5 exposure induced serious bad effects shown as inflammation and oxidative stress in hyperlipidemic rats, then triggered cardiomyocyte apoptosis by caspase3 through JNK/P53 pathway 33. Along with the cardiomyocyte, Zhang et al. indicated that PM2.5 could activate inflammatory axis of COX-2/PGES/PGE2 in vascular endothelial cells to promote cell apoptosis and inflammatory response 34. While Wang et al. revealed PM2.5 could activate the p53-bax-caspase pathway to induce endothelial cell apoptosis in cardiovascular diseases 35. Lv et al. also confirmed PM2.5 served as an important role in cardiac injury by enhancing intracellular ROS production and activating the MAPKs signaling pathway 36. In the reproductive system, Hu et al. indicated that PM2.5 was toxic to induce apoptosis in hESCs by inhibition of ROS-mediated Nrf2 pathway activity 37. And Peng et al. indicate that PM2.5 induced embryo toxicity by ROS-JNK/ERK apoptosis pathway resulting in adverse pregnancy outcomes 38. In the respiratory system, our team has confirmed that PM2.5 aggravated apoptosis in cigarette-inflamed bronchial epithelial cells, and the responses could be suppressed by Z-VAD-FMK by caspase3 39. Then, we found that PM2.5 could down-regulate miR-194-3p and accelerate apoptosis in this cigarette-inflamed bronchial epithelium by targeting DAPK1 40. Li et al. and Jiao et al. both found PM2.5 lead to the mitochondrial structure disorder via ROS generation to incur bronchial epithelial cell apoptosis, which resulted in respiratory damage 41, 42. In addition to the above ROS signaling pathways, Qi et al. have suggested that PM2.5 could result in apoptosis and cell damage by activating MAPK/NF-кB/STAT1 pathway in A549 cells 43. Excepting the above pathways, Shirali et al. have given a thought into PM2.5 exposure induced apoptosis via the activation of TNF-a pathway in human lung epithelial cells (L132) cells 44. In skin, Guan et al. indicated that PM2.5 induced DNA damage and mitochondria-dependent apoptosis in a dose-dependent manner in human keratinocyte (HaCaT) cells to induce skin irritation and damage 45. In central nervous system, PM2.5 altered the protein expression of apoptosis-related markers (mainly as bax and bcl-2), activated caspase-3 and caused neuronal apoptosis and synaptic injuries 46.

From the above studies in different systems, we could discover that autophagy-related diseases induced by PM2.5 are mainly involved in inflammation-related signaling pathways, including β2AR/PI3K/Akt, caspase3/JNK/P53, COX-2/PGES/PGE2, ROS-JNK/ERK, MAPK/NF-кB/STAT1 (Figure 1). That is to say, the inflammatory response caused by PM2.5 is the main precursor of apoptosis. Blocking the related inflammatory pathway will become the promising target for the treatment and prevention of PM2.5 related diseases in the future.

## PM2.5 and pyroptosis

As is known, programmed cell death (PCD) is on behalf of the way in which cells die depending on specific genes encoding signals or activities, including apoptosis, autophagy, and pyroptosis. Above are the two kinds of programmed cell death, now the third one, pyroptosis. Pyroptosis is found to be a new procedural and inflammatory death that pyroptotic cells undergo nuclear condensation and chromatin DNA fragmentation 47. During pyroptosis process, the pores formed on the cell membrane disrupts the balance of ion gradients on both side of the cell, leading to water inflow, cell swelling, cell membrane rupture, and proinflammatory mediators release, including IL- 1β, IL-18^48^.

Partial lysosome breakage caused by inorganic particles is related to the induction of inflammasome and caspase-1, while they may eventually cause cell death through pyroptosis. Research showed the role of NLRP3 inflammasome activation served as a key step in pyroptosis, so we focused on PM2.5-induced NLRP inflammasome activation. In the circulatory system, Duan et.al found that PM2.5 exposure could increase the protein expression of NLRP3 activation-associated markers. The markers contain NLRP3, IL-1β, IL-18, Cleaved caspase-1 p10, while Cleaved IL-1β was upregulated in heart tissue of PM-induced mice. The results mean that PM2.5 exposure could induce structural and functional disorders, which might be linked to the NLRP3 inflammasome activation 49. In an in vitro study, Shen et.al have proved that cooking oil fumes-derived PM2.5 could reduce HUVECs viability, induce the overproduction of ROS, which could affect the blood vessel formation through ROS-mediated NLRP3 inflammasome pathway^50^. In the central nervous system, Chu et.al revealed that the neurotransmitters were significantly disorder, while some metallic substances could deposit there. And the NLRP3 inflammasome was more activated after PM2.5 exposure, which suggested NLRP3 signaling pathway might play a critical role in depression induced by ambient PM2.5 51. In addition to depression, the role of PM2.5 induced NLRP3-mediated pyroptosis in Alzheimer's disease should not be underestimated. Wang et al. pointed out that in the in vitro model of Alzheimer's disease, PM2.5 exposure aggravated oAβ-induced neuronal damage and inflammatory response in neuron-microglial co-culture by increasing IL-1β produced through activation of NLRP3 inflammasome 3.

Pyroptosis, also known as cell inflammatory necrosis, is a kind of programmed cell death, which is characterized by the continuous expansion of cells until the rupture of the cell membrane, leading to the release of cell contents and activating a strong inflammatory response by a large number of inflammatory factors. Pyroptosis is an important innate immune response of the body mediated by gasdermin D, which plays an important role in the fight against infection. However, the exact mechanism of pyroptosis in PM2.5-induced lung diseases remains to be further explored.

### PM2.5 and ferroptosis

Ferroptosis is a new confirmed type of programmed cell death, which is apart from apoptosis, necrosis and autophagy. The main mechanism of ferroptosis is that under the action of divalent iron or ester oxygenase, it catalyzes the high expression of unsaturated fatty acids on the cell membrane, resulting in liposome peroxidation, thus inducing cell death 47. Accumulating evidence indicates that cell death related to cardiomyopathy, tumorigenesis and cerebral hemorrhage has an important relationship with ferroptosis 52. However, PM2.5 related ferroptosis is still rare.

### Summary and question

Ensuring the dynamic balance of cell survival and death is critical for normal development and homeostasis, and also for preventing diseases. Conventional novel cell death pathways include apoptosis and necrosis, while new types of regulated cell death include necroptosis, pyroptosis and ferroptosis. And PM2.5 was involved in various diseases related to different system in body through the above ways of cell death, especially in the respiratory system, circulatory system, central nervous system and reproductive system. However, how to prevent the harm of PM2.5 to the major systems of the body, and how to reverse the various ways of cell death induced by PM2.5 are worthy of our further exploration.
